# Abnormal chromosome behavior during meiosis in the allotetraploid of *Carassius auratus red var.* (♀) × *Megalobrama amblycephala* (♂)

**DOI:** 10.1186/s12863-014-0095-6

**Published:** 2014-09-02

**Authors:** Qinbo Qin, Yude Wang, Juan Wang, Jing Dai, Yun Liu, Shaojun Liu

**Affiliations:** 1 Key Laboratory of Protein Chemistry and Developmental Biology of the State Education Ministry of China, College of Life Sciences, Hunan Normal University, Changsha 410018, People’s Republic of China

**Keywords:** Allotetraploid, Chromosome behavior, FISH, Gamete

## Abstract

**Background:**

Allopolyploids generally undergo bivalent pairing at meiosis because only homologous chromosomes pair up. On the other hand, several studies have documented abnormal chromosome behavior during mitosis and meiosis in allopolyploids plants leading to the production of gametes with complete paternal or maternal chromosomes. Polyploidy is relatively rare in animals compared with plants; thus, chromosome behavior at meiosis in the allopolyploid animals is poorly understood.

**Results:**

Tetraploid hybrids (abbreviated as 4nRB) (4n = 148, RRBB) of *Carassius auratus red var.* (abbreviated as RCC) (2n = 100, RR) (♀) × *Megalobrama amblycephala* (abbreviated as BSB) (2n = 48, BB) (♂) generated gametes of different size. To test the genetic composition of these gametes, the gynogenetic offspring and backcross progenies of 4nRB were produced, and their genetic composition were examined by chromosome analysis and FISH. Our results suggest that 4nRB can produce several types of gametes with different genetic compositions, including allotetraploid (RRBB), autotriploid (RRR), autodiploid (RR), and haploid (R) gametes.

**Conclusions:**

This study provides direct evidence of abnormal chromosome behavior during meiosis in an allotetraploid fish.

## Background

Polyploids are reported in plants, fish and amphibians, and are usually fit and well adapted [1],[2]. Most polyploids have an even number of chromosomes sets, with four being the most common (tetraploidy). Allopolyploids result from the combination of chromosome sets from two or more different taxa that undergo bivalent pairing at meiosis because only homologous chromosomes pair up [3],[4]. It is important that a diploid-like pairing system prevents meiotic irregularities and improves the efficiency of gamete production in allopolyploid species [5].

Allopolyploid speciation can result from chromosome doubling in a diploid hybrid to create unreduced gametes. When these diploid eggs and sperm are fertilized, they produce a tetraploid [6],[7]. In our previous study, the tetraploid (abbreviated as 4nRB) (4n = 148, RRBB) was obtained in the first generation of *Carassius auratus red var.* (abbreviated as RCC) (2n = 100, RR) (♀) × *Megalobrama amblycephala* (abbreviated as BSB) (2n = 48, BB) (♂), and resulted from the inhibition of the first cleavage of the fertilized eggs [8]–[10]. In this study, we provide direct evidence that abnormal chromosome behavior during meiosis occur in the allotetraploid hybrids, but bivalent pairing and the mechanisms of the abnormal chromosome behavior need to be investigated in future. This is the first report of abnormal chromosome behavior during meiosis in allotetraploid fish, and will contribute to the understanding of vertebrate polyploidization and evolution.

## Methods

All samples were cultured in ponds at the Protection Station of Polyploidy Fish, Hunan Normal University, and fed with artificial feed. Fish treatments were carried out according to the regulations for protected wildlife and the Administration of Affairs Concerning Animal Experimentation, and approved by the Science and Technology Bureau of China. Approval from the Department of Wildlife Administration was not required for the experiments conducted in this paper. The fish were deeply anesthetized with 100 mg/L MS-222 (Sigma-Aldrich, St Louis, MO, USA) before dissection.

### Crosses

During the reproductive seasons (April to June) in 2004, 2005, and 2006, 4nRB of RCC (♀) × BSB (♂) were produced. During the reproductive seasons of 2006 and 2007, gynogenetic offspring (G-1, G-2, G-3) were obtained by artificial gynogenesis from 4nRB eggs that were activated with UV-treated sterilized BSB sperm, without chromosomes doubling treatment. During the reproductive season of 2008, the backcross progenies (H-1, H-2, H-3) of 4nRB (♀) × RCC (♂) were produced.

### Spermatozoa phenotype

The semen of 4nRB was collected with a clean pipette and transferred into 2.5% glutaraldehyde solution. The semen was centrifuged at 2000 r/min for 1 min, fixed in 4% glutaraldehyde solution overnight, and then fixed in 1% osmic acid solution for 2 h. The spermatozoa were dehydrated in alcohol, dropped onto slides, desiccated, coated atomized gold, and then observed with an X-650 (Hitachi) SEM scan-electron micro-scope.

### Preparation of chromosome spreads

To determine ploidy, chromosome counts were performed using kidney tissue from 10 individuals each of RCC, BSB, G-1, G-2, G-3, H-1, H-2, and H-3 at 1 year of age. After culture for 1–3 d at a water temperature of 18–22°C, the samples were injected with concanavalin one to three times at a dose of 2–8 mg/g body weight. The interval between injections was 12–24 h. Six hours prior to dissection each sample was injected with colchicine at a dose of 2–4 mg/g body weight. The kidney tissue was ground in 0.9% NaCl, followed by hypotonic treatment with 0.075 m KCl at 37°C for 40–60 min and then fixed in 3:1 methanol–acetic acid with three changes. The cells were dropped onto cold, wet slides and stained for 30 min in 4% Giemsa. The shape and number of chromosomes were analyzed under a microscope. For each type of fish, 200 metaphase spreads (20 metaphase spreads from each sample) of chromosomes were analyzed. The preparations were examined under an oil lens at a magnification of 3330 ×.

### Fluorescence *in situ* hybridization

Species-specific centromere probes of fluorescence in situ hybridization (FISH) were made from RCC and amplified by PCR using the primers 5’-TTCGAAAAGAGAGAATAATCTA-3’ and 5’-AACTCGTCTAAACCCGAACTA-3’. The FISH probes were produced by Dig-11-dUTP labeling (using a nick translation kit, Roche, Germany) of purified PCR products. FISH was performed according to He et al. [11]. For each type of fish, 200 metaphase chromosome spreads from 10 individuals were analyzed under a Leica inverted CW4000 microscope with a Leica LCS SP2 confocal imaging system (Leica, Germany). Captured images were colored and overlapped in Adobe Photoshop CS6.

## Results

### The size of gametes produced by 4nRB

**Figure 1:**
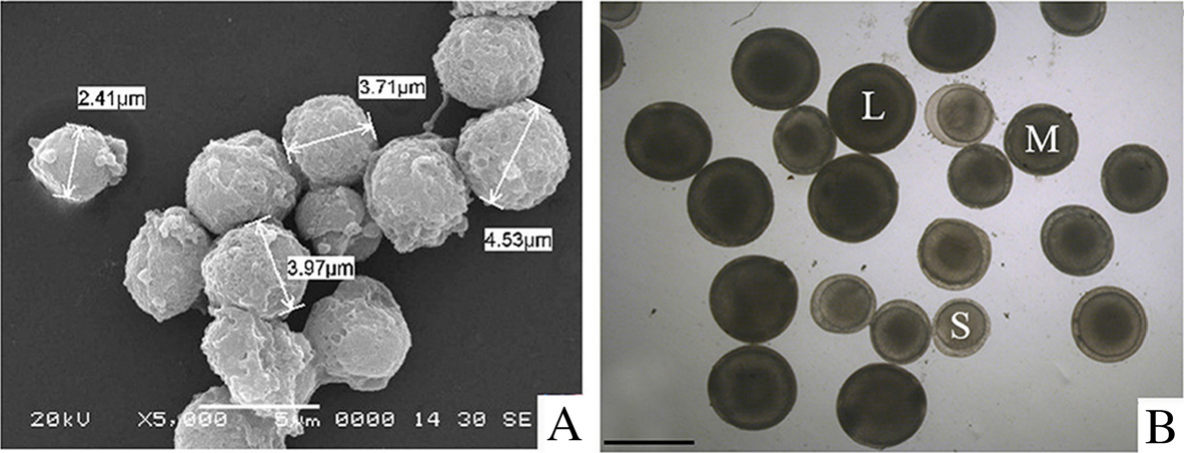
**Gamete of 4nRB. A**: The male 4nRB hybrids produced spermatozoon with different sizes; **B**: The female 4nRB hybrids produced three sizes of eggs, The average diameter of the larger eggs(L) was 0.20 cm, the average diameter of the medium eggs(M) was 0.17 cm, and the average diameter of the small eggs (S) was 0.13 cm; Bar is 2.0 cm.

The male 4nRB hybrids produced different sizes of spermatozoa. The large-size spermatozoa comprised 38% of the total, with an average diameter of 4.5 μm. The medium-size spermatozoa accounted for 56%, with an average diameter of 3.8 μm, while the small-size spermatozoa with an average diameter of 2.4 μm made up 6% (Figure 1A). In addition, the female 4nRB hybrids produced three sizes of eggs. The large-size eggs accounted for 89% of the total, with an average diameter of 0.20 cm. The medium-size eggs with an average diameter of 0.17 cm accounted for 7%, and the small-size eggs with an average diameter of 0.13 cm comprised 4% (Figure 1B).

### Formation of gynogenetic and backcross progenies

**Figure 2:**
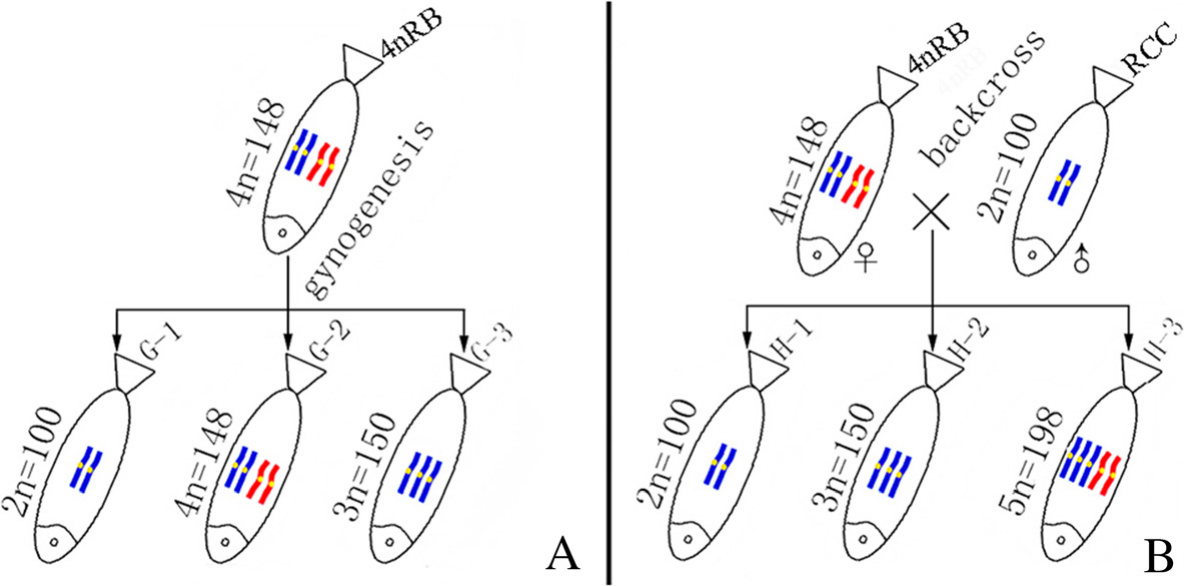
**Formation of experimental fish. A**: The gynogenetic progenies of 4nRB; **B**: The backcross progenies of 4nRB; the parental origin of chromosomes was marked by blue and red.

From the cross of RCC (♀) × BSB (♂), we obtained 4nRB. Without chromosome doubling treatment, the fertilized eggs of 4nRB developed into normal live gynogenetic progenies (G-1, G-2, and G-3) after activation with UV-irradiated BSB sperm (Figure 2A). Backcross progenies (H-1, H-2, and H-3) of 4nRB (♀) × RCC (♂) were also produced (Figure 2B).

### Examination of chromosome number

**Figure 3:**
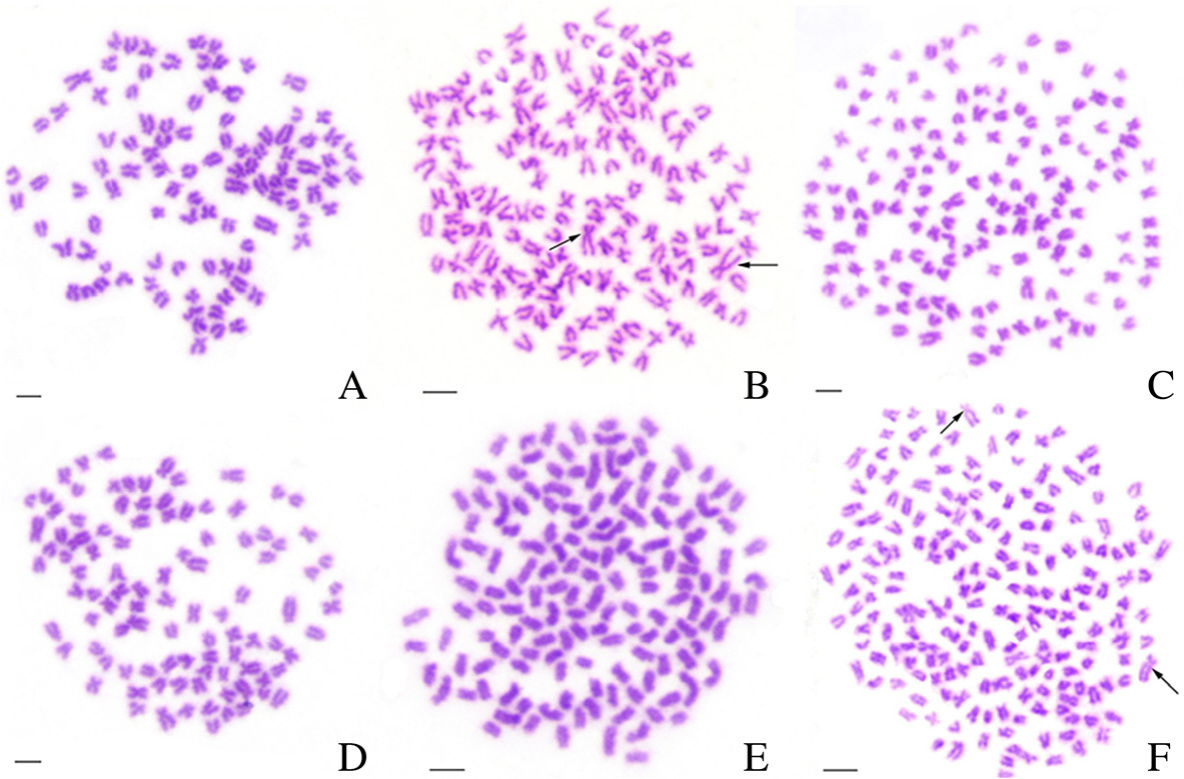
**Chromosome spreads at metaphase in the gynogenetic offspring (G-1, G-2, and G-3) and backcross progenies (H-1, H-2, and H-3) of 4nRB. A**: The 100 chromosomes of G-1, in which no largest submetacentric chromosome was found; **B**: The 148 chromosomes of G-2, in which a pair of the largest submetacentric chromosomes (arrows) were indicated; **C**: The 150 chromosomes of G-3, in which the largest submetacentric chromosomes from BSB was absent; **D**: The 100 chromosomes of H-1, in which the largest submetacentric chromosome from BSB was absent; **E**: The 150 chromosomes of H-2, in which the largest submetacentric chromosome from BSB was absent; **F**: The 198 chromosomes of H-3, in which a pair of the largest submetacentric chromosomes (arrows) were indicated; Bar in A–D, 3 μm.

Table 1 illustrates the distribution of chromosome numbers in RCC, BSB, 4nRB, G-1, G-2, G-3, H-1, H-2, and H-3. Among the RCC samples, 90% of metaphase spreads had 100 chromosomes. Among the BSB samples, 87.5% of metaphase spreads possessed 48 chromosomes (Table 1). A pair of the largest submetacentric chromosomes were observed in BSB that could be used as a chromosomal marker to identify this species. Among the RCC chromosomes, there was no evidence of a special largest submetacentric chromosome. In the 4nRB samples, 78% of metaphase spreads had 148 chromosomes, among which a pair of the largest submetacentric chromosomes derived from BSB were observed [8]. In G-1 samples, 81% of metaphase spreads had 100 chromosome, among which the largest submetacentric chromosomes from BSB was absent (Figure 3A). In the G-2 samples, 86% of metaphase spreads had 148 chromosomes, and a pair of the largest submetacentric chromosomes derived from BSB were observed (Figure 3B). In G-3 samples, 84% of metaphase spreads had 150 chromosomes, among which the largest submetacentric chromosome from BSB was absent (Figure 3C). In the H-1 samples, 86.5% of metaphase spreads had 100 chromosomes, among which the large submetacentric chromosome from BSB was absent (Figure 3D). In the H-2 samples, 84.5% of metaphase spreads had 150 chromosomes, in which the largest submetacentric chromosomes from BSB was absent (Figure 3E). Finally, in the H-3 samples, 80.5% of metaphase spreads had 198 chromosomes, among which the pair of a largest submetacentric chromosomes from BSB were observed (Figure 3F).

**Table 1:** Examination of chromosome number in RCC, BSB, 4nRB, gynogenetic offspring (G-1, G-2, and G-3) and backcross progenies (H-1, H-2, and H-3) of 4nRB

**Fish type**	**No. of metaphase**	**Distribution of chromosome number**									
		**<48**	**48**	**<100**	**100**	**<148**	**148**	**<150**	**150**	**<198**	**198**
RCC	200			20	180						
BSB	200	25	175								
4nRB	200					44	156				
G-1	200			38	162						
G-2	200					28	172				
G-3	200							32	168		
H-1	200			27	173						
H-2	200							31	169		
H-3	200									39	161

### Fluorescence in situ hybridization

**Figure 4:**
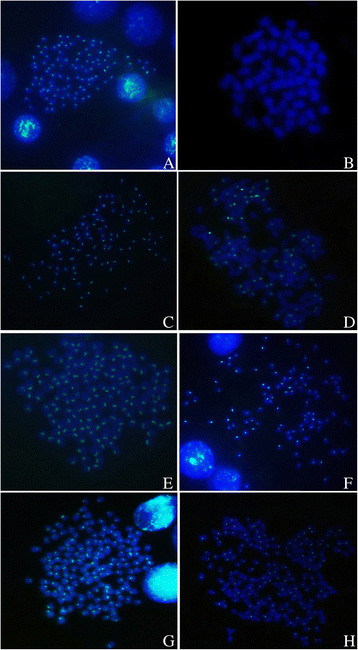
**Examination of hybridizing signals by FISH in RCC, BSB, the gynogenetic offspring (G-1, G-2, and G-3) and backcross progenies (H-1, H-2, and H-3) of 4nRB. A**: The centromere probe hybridized to 100 chromosomes in RCC; **B**: No chromosome of BSB was hybridized; **C**: The centromere probe hybridized to 100 chromosomes of G-1; **D**: The centromere probe hybridized to 100 RCC-derived chromosomes in G-2; **E**: The centromere probe hybridized to 150 chromosomes of G-3; **F**: The centromere probe hybridized to 100 chromosomes of H-1; **G**: The centromere probe hybridized to 150 chromosomes of H-2; **H**: The centromere probe hybridized to 150 RCC-derived chromosomes in H-3.

The species-specific centromere probe (repetitive sequences of 263 bp; sequence number JQ086761) hybridized to all 100 chromosomes in RCC (Figure 4A) but none in BSB (Figure 4B). Thus, RCC and BSB-derived chromosomes could be discriminated by FISH using the centromere probe. The probe was hybridized to the metaphase chromosomes of the gynogenetic offspring and backcross progenies of 4nRB; the results were shown in Table 2. Among the G-1 samples, the centromere probe hybridized to 100 chromosomes in 94.5% of metaphase spreads (Figure 4C), suggesting that G-1 possessed two sets of RCC-derived chromosomes. Among the G-2 samples, the probe hybridized to 100 chromosomes in 90.5% of metaphase spreads (Figure 4D), indicating that G-2 had two sets of RCC-derived chromosomes. The probe hybridized to 150 chromosomes in 87% of metaphase spreads of G-3 (Figure 4E), suggesting that G-3 possessed three sets of RCC-derived chromosomes. Among the H-1 samples, the probe hybridized to 100 chromosomes in 91% of metaphase spreads (Figure 4F), suggesting that two sets of RCC-derived chromosomes were present. In H-2, the probe hybridized to 150 chromosomes in 84% of metaphase spreads (Figure 4G), suggesting that H-2 possessed three sets of RCC-derived chromosomes. In H-3, the probe hybridized to 150 chromosomes in 80.5% of metaphase spreads (Figure 4H), indicating that H-3 had three sets of RCC-derived chromosomes.

**Table 2:** Examination of hybridizing signals by FISH in RCC, BSB, the gynogenetic offspring (G-1, G-2, and G-3) and backcross progenies (H-1, H-2, and H-3) of 4nRB

**Fish type**	**No. of fish**	**No. of metaphase**	**Distribution of chromosome loci number**					
			**<48**	**48**	**<100**	**100**	**<150**	**150**
RCC	10	200			15	185		
BSB	10	200	0	0				
G-1	10	200			11	189		
G-2	10	200			19	181		
G-3	10	200					26	174
H-1	10	200			18	182		
H-2	10	200					32	168
H-3	10	200					39	161

## Discussion

Generally, the pairing of homologous chromosomes is defective in the F_1_ hybrids because of divergence in the structure and number of chromosomes [6]. However, the F_1_ hybrids can generate unreduced gametes by chromosome doubling; thus, they can produce allotetraploid offspring after fertilization of the diploid eggs and sperm from females and males of the diploid hybrid. This produces an allotetraploid in which the two homologous chromosome sets pair independently and allodiploid gametes are created [3],[6],[9]. In our previous study, allotetraploid hybrids (4nRB, 4n = 148, RRBB) were obtained in the first generation of RCC (2n = 100, RR, ♀) × BSB (2n = 48, BB, ♂), and possessed two sets of RCC-derived chromosomes and two sets of BSB-derived chromosomes [8],[9]. Theoretically, the two homologous chromosomes sets should pair independently, and thus bring about diploid-like meiotic behavior in 4nRB to produce allodiploid gametes (2n = 74, RB). However, the genetic composition of gamete indicated the surprising proof of abnormal chromosome behavior during meiosis in 4nRB.

In this paper, gynogenetic offspring (G-1, G-2, and G-3) were obtained by artificial gynogenesis, from 4nRB eggs that were activated with UV-treated sterilized sperm of BSB (2n = 48) but not subjected to chromosome doubling treatment (Figure 2C). The backcross progenies (H-1, H-2, and H-3) of 4nRB (♀) × RCC (♂) were then produced (Figure 2B). We evaluated the genetic composition of the gynogenetic offspring and backcross progenies by analyzing chromosome numbers and loci to infer chromosome behavior during meiosis in 4nRB, including the ploidy level and genetic composition of the gametes. For the gynogenetic offspring, our results suggested that G-1 (2n = 100, RR) were autodiploids with two sets of RCC-derived chromosomes (Figure 3A; Figure 4C), G-2 (4n = 148, RRBB) were allotetraploid with two sets of RCC-derived chromosomes and two sets of BSB-derived chromosomes (Figure 3B; Figure 4D), and G-3 (3n = 150, RRR) were autotriploids with three sets of RCC-derived chromosomes (Figure 3C; Figure 4E). Thus, these results provide direct proof of that 4nRB can produce many gametes with different genetic compositions, including allotetraploid (RRBB), autotriploid (RRR), and autodiploid (RR) gamete. In the backcross progenies, H-1 were autodiploids with two sets of RCC-derived chromosomes (Figure 3D; Figure 4F), suggesting that 4nRB can produce the haploid gamete (R). H-2 were autotriploids with three sets of RCC-derived chromosomes (Figure 3E; Figure 4G), suggesting that 4nRB can produce the autodiploid gametes (RR). Finally, H-3 (5n = 198, RRRBB) were allopentaploids with three sets of RCC-derived chromosomes and two sets of BSB-derived chromosomes (Figure 3F; Figure 4H), suggesting that 4nRB can produce the allotetraploid gametes (RRBB).

In 1935, the separation of parental genomes during mitotic and meiotic divisions of hybrid cells was firstly proposed in sexual hybrids between cultivated *Brassica* species [12]. Until now, several studies have documented abnormal chromosome behavior during mitosis and meiosis in allopolyploids that leads to the production of gametes with complete paternal or maternal chromosomes [13]–[15]. In this paper, 4nRB produce several types of gametes with different genetic compositions, including allotetraploid (RRBB), autotriploid (RRR), autodiploid (RR), and haploid (R) gametes. On the basis of genetic composition of gamete, we speculate that some germ cells may perform the chromosome doubling by premeiotic endoreduplication, endomitosis, or fusion of oogonia germ in 4nRB [8],[9], some of which show normal chromosome behavior (homologous chromosomes sets pair independently) during meiosis and developed into unreudced allotetraploid gametes, but other part of which show complete separation of the parental genomes, but not normal chromosome behavior during meiosis and develop into gametes with one or more RCC-derived chromosome sets. Of course, theoretically, other types of gametes with one or more sets of BSB-derived chromosomes may also have been produced because of complete separation of the parental genomes during meiosis, but were not detected in our study.

## Conclusions

Diploid hybrid embryos (RB) of *Carassius auratus red var.* (2n = 100, RR, ♀) × *Megalobrama amblycephala* (2n = 48, BB, ♂) developed into surviving allotetraploid offspring (4nRB, RRBB) by somatic chromosome doubling [8],[9]. However, abnormal chromosome behavior during meiosis occurred in the allotetraploid fish to form gametes with different genetic compositions. This paper is the first detailed reports of abnormal chromosome behavior during meiosis in allotetraploid fish. Importantly, 4nRB is a significant experimental material for study of vertebrate chromosome evolution, and can provide an abundant gamete source for the production of other diploids or polyploids fish.

## Competing interests

The authors declare that they have no competing interests.

## Authors’ contributions

QBQ, SJL and YL designed the experiments; YDW and JD performed the experiments; QBQ performed the statistical analysis and wrote the manuscript. All authors read and approved the final manuscript.
